# Short CDRL1 in intermediate VRC01-like mAbs is not sufficient to overcome key glycan barriers on HIV-1 Env

**DOI:** 10.1128/jvi.00744-24

**Published:** 2024-09-06

**Authors:** Parul Agrawal, Maria L. Knudsen, Anna MacCamy, Nicholas K. Hurlburt, Arineh Khechaduri, Kelsey R. Salladay, Gargi M. Kher, Latha Kallur Siddaramaiah, Andrew B. Stuart, Ilja Bontjer, Xiaoying Shen, David Montefiori, Harry B. Gristick, Pamela J. Bjorkman, Rogier W. Sanders, Marie Pancera, Leonidas Stamatatos

**Affiliations:** 1 Vaccine and Infectious Disease Division, Fred Hutchinson Cancer Center, Seattle, Washington, USA; 2 Department of Medical Microbiology and Infection Prevention, Amsterdam UMC, University of Amsterdam, Amsterdam, the Netherlands; 3 Amsterdam Institute for Infection and Immunity, Infectious Diseases, Amsterdam, the Netherlands; 4 Division of Surgical Sciences, Duke University, Durham, North Carolina, USA; 5 California Institute of Technology, Pasadena, California, USA; 6 Department of Microbiology and Immunology, Weill Medical College of Cornell University, New York, New York, USA; 7 Vaccine Research Center, National Institutes of Allergy and Infectious Diseases, NIH, Bethesda, Maryland, USA; 8 Department of Global Health, University of Washington, Seattle, Washington, USA; Icahn School of Medicine at Mount Sinai, New York, New York, USA

**Keywords:** HIV-1, VRC01-class antibodies, CDRL1, neutralization, BCR sequencing

## Abstract

VRC01-class broadly neutralizing antibodies (bnAbs) have been isolated from people with HIV-1, but they have not yet been elicited by vaccination. They are extensively somatically mutated and sometimes accumulate CDRL1 deletions. Such indels may allow VRC01-class antibodies to accommodate the glycans expressed on a conserved N276 N-linked glycosylation site in loop D of the gp120 subunit. These glycans constitute a major obstacle in the development of VRC01-class antibodies, as unmutated antibody forms are unable to accommodate them. Although immunizations of knock-in mice expressing human VRC01-class B-cell receptors (BCRs) with specifically designed Env-derived immunogens lead to the accumulation of somatic mutations in VRC01-class BCRs, CDRL1 deletions are rarely observed, and the elicited antibodies display narrow neutralizing activities. The lack of broad neutralizing potential could be due to the absence of deletions, the lack of appropriate somatic mutations, or both. To address this point, we modified our previously determined prime-boost immunization with a germline-targeting immunogen nanoparticle (426c.Mod.Core), followed by a heterologous core nanoparticle (HxB2.WT.Core), by adding a final boost with a cocktail of various stabilized soluble Env trimers. We isolated VRC01-like antibodies with extensive somatic mutations and, in one case, a seven-amino acid CDRL1 deletion. We generated chimeric antibodies that combine the vaccine-elicited somatic mutations with CDRL1 deletions present in human mature VRC01 bnAbs. We observed that CDRL1 indels did not improve the neutralizing antibody activities. Our study indicates that CDRL1 length by itself is not sufficient for the broadly neutralizing phenotype of this class of antibodies.

**IMPORTANCE:**

HIV-1 broadly neutralizing antibodies will be a key component of an effective HIV-1 vaccine, as they prevent viral acquisition. Over the past decade, numerous broadly neutralizing antibodies (bnAbs) have been isolated from people with HIV. Despite an in-depth knowledge of their structures, epitopes, ontogenies, and, in a few rare cases, their maturation pathways during infection, bnAbs have, so far, not been elicited by vaccination. This necessitates the identification of key obstacles that prevent their elicitation by immunization and overcoming them. Here we examined whether CDRL1 shortening is a prerequisite for the broadly neutralizing potential of VRC01-class bnAbs, which bind within the CD4 receptor binding site of Env. Our findings indicate that CDRL1 shortening by itself is important but not sufficient for the acquisition of neutralization breadth, and suggest that particular combinations of amino acid mutations, not elicited so far by vaccination, are most likely required for the development of such a feature.

## INTRODUCTION

VRC01-class broadly neutralizing HIV-1 antibodies have been isolated from several people living with HIV-1 infected by different HIV-1 viruses and bind a well-defined epitope within the CD4-binding site (CD4-BS) (1–6). Antibodies in this class are all derived from the pairing of VH1-2*02 heavy chains (HCs) with a limited number of light chains (LCs) expressing rare five-amino acid (5-aa) CDRL3s (7). VRC01-class bnAbs isolated during infection (referred to as “mature,” m, antibodies) are extensively mutated in both their HCs and LCs and can be up to 50% divergent in their amino acid sequences. Nevertheless, they share similar structures and engage their epitope in a similar manner (4–6). Contrary to most other anti-CD4-BS antibodies, they recognize the CD4-BS primarily through their gene-encoded CDRH2 domains (5, 6, 8, 9), although their CDRH3s also contribute to this interaction (10, 11). VRC01-class bnAbs have been shown to prevent infection of humanized mice by HIV-1 and of non-human primates by S(H)IV (12–14). Importantly, the first VRC01-class antibody isolated (VRC01) prevented HIV-1 acquisition from susceptible viruses in two phase 3 clinical trials (HVTN 703/704) (15).

Although the VRC01-class bnAbs isolated from HIV-1-infected individuals bind to diverse HIV-1 Env proteins and neutralize diverse HIV-1 isolates, their unmutated precursors (referred to as “germline,” gl) do neither (16–20). In fact, Envs derived from circulating viruses capable of binding glVRC01-class antibodies are presently not known. As a result, during immunizations with such Envs, naïve B cells expressing glVRC01-class B-cell receptors (BCRs) do not become activated (21), and hence, VRC01-class bnAbs have not yet been elicited through vaccination.

The key obstacles preventing glVRC01-class antibodies from binding Envs are as follows: (i) glycans expressed on the conserved N-linked glycosylation site (NLGS) at position 276 (in loop D), (ii) glycans present on the V5 region of Env, and (iii) the length and glycosylation of the V1–V3 Env regions (16, 19, 20, 22). Consequently, specifically designed Env-derived proteins capable of engaging glVRC01-class antibodies (“gl-targeting” Envs) and their corresponding BCRs have been generated (16, 19, 20, 22, 23).

As wild-type (WT) animal species do not express orthologs of the human VH1-2*02 allele (7), transgenic mice engineered to express different forms of glVRC01-class BCRs have been developed and are routinely employed to test immunogens and immunization schemes that would elicit VRC01-class bnAbs (19, 24–32). One such knock-in (KI) mouse is heterozygous for the gl HC of VRC01 mAb (VRC01^glHC^) but expresses diverse endogenous mouse LCs (28). In total, approximately 0.08% of naïve B cells in these mice express glVRC01-like BCRs (28, 33, 34). We have reported that a prime immunization with the gl-targeting clade C 426c.Mod.Core immunogen (expressed as self-assembling nanoparticles) followed by a booster immunization with self-assembling nanoparticles expressing the heterologous clade B HxB2.WT.Core immunogen (which does not activate naïve glVRC01 B cells) results in the elicitation of partially mutated VRC01-like B cells and antibody responses in these KI mice (30–32). The vast majority of these antibodies express the mouse κ8–30*01, which has a CDRL1 of 17 aa in length. In contrast, mVRC01-class bnAbs generated during HIV-1 infection typically have indels in their CDRL1 (6). A shorter CDRL1 (7- to 11-aa long) is believed to be the reason why mVRC01-class bnAbs can bypass the glycans present at the conserved position N276 of the gp120 subunit and neutralize diverse HIV-1 strains. Indeed, CDRL1 modifications (along with modifications in LFWR3 and LCDR3, associated with adaptations to N276 glycans and Loop D contacting) appear toward the end of the maturation process of VRC01-class antibodies during HIV-1 infection (35).

Here, we employed a prime-boost immunization schema in the VRC01^glHC^ mouse model that led to the isolation of a VRC01-like antibody whose κ8–30*01 LC had acquired a CDRL1 indel. The neutralizing potential of this antibody, however, was not broad and did not lead to neutralization of tier 2 HIV-1 viruses. In parallel, by replacing the long CDRL1 of partially mutated VRC01-like antibodies elicited during this immunization with the much shorter CDRL1 present on the human mVRC01-class bnAb VRC01 (mVRC01), we confirmed that CDRL1 indels did not improve their neutralizing potentials. Collectively, therefore, our data indicate that a shorter CDRL1 is important but not sufficient for improving the neutralizing properties of these elicited VRC01-like antibodies.

## RESULTS

### Serological analysis during immunization

**Fig 1:**
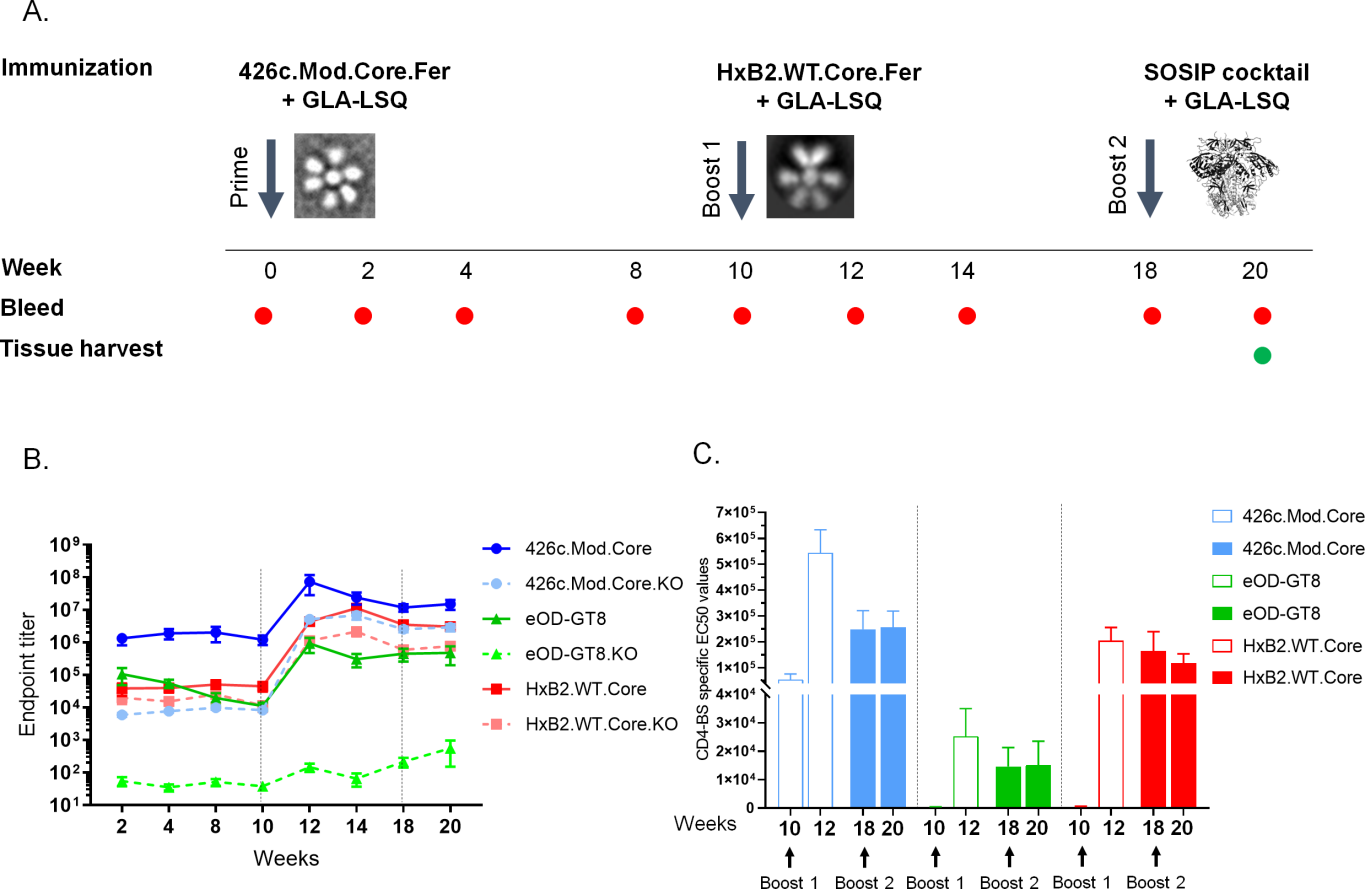
Antibody responses 2 weeks following the final boost immunization. (**A**) Mice (*n* = 5) were primed with 426c.Mod.Core ferritin nanoparticles with GLA-LSQ at week 0, followed by adjuvanted HxB2.WT.Core ferritin nanoparticle boost at week 10, and adjuvanted SOSIP cocktail as the final boost at week 18. Mice were bled at the indicated time points (red circles) and sacked for blood, spleen, and lymph node tissues, at week 20 (green circle). (**B**) Plasma was assayed by ELISA for binding against 426.Mod.Core (blue solid line), eOD-GT8 (green solid line), HxB2.WT.Core (red solid line), as well as their corresponding antigens with CD4-BS or VRC01 epitope knock-out (KO) (dotted lines); mean endpoint titers against the indicated proteins with SEM values are shown over time. Black dotted lines indicate the time of the booster immunizations. (**C**) CD4-BS-specific EC50 values against the indicated proteins are shown post each boost where open bars represent response post Boost 1 and filled bars, post Boost 2. See also Table S1 and Fig. S1.

A prime immunization of VRC01^glHC^ KI mice with the clade C-derived 426c.Mod.Core self-assembling nanoparticles (see Materials and Methods and [19] for details) followed by a boost immunization with the clade B-derived HxB2.WT.Core self-assembling nanoparticles results in the development of partially mutated VRC01-like antibodies that can neutralize the autologous 426c virus whose Env artificially lacks the N276 NLGS (426c single mutant, 426c.SM) but not the 426c virus expressing the WT fully glycosylated Env (426c.WT) (31). To improve the maturation of these antibodies, here, we examined whether adding a second boost immunization (following the HxB2.WT.Core immunization), with a cocktail of 11 heterologous soluble, stabilized Env trimers (SOSIP) (Table S1), will lead to the elicitation of VRC01-like antibodies that more efficiently accommodate the N276-associated glycans on functional Envs (Fig. 1A). One reason for employing SOSIP immunogens as final boost was that SOSIPs may select VRC01-like BCRs with mutations that allow them to engage the VRC01 epitope as present on functional Envs. Another reason for using SOSIP booster immunogens after the two immunizations with core gp120s was to avoid the activation of B cells targeting epitopes present only on the cores, which are occluded in native Envs.

In agreement with our previous observations, immunization with 426c.Mod.Core nanoparticles elicited robust autologous plasma antibody responses (Fig. 1B, blue line; Fig. S1A), the majority of which targeted the CD4-BS on that Env (Fig. S1B, blue line) as demonstrated by the reduced binding to the CD4-BS knock-out (KO) version (D368R/E370A/D279A) of this protein (Fig. 1B). These antibodies also recognized the heterologous glVRC01-targeting protein eOD-GT8 ((16); Fig. 1B, green line; Fig. S1A) in a CD4-BS-dependent manner (Fig. S1B, green line). All animals generated durable anti-HxB2.WT.Core plasma antibody responses (Fig. 1B, red line; Fig. S1A), a fraction of which targeted the CD4-BS on that Env (Fig. S1B, red line). The boost immunization with HxB2.WT.Core nanoparticles increased the plasma antibody responses against all proteins tested (Fig. 1B, post week 10), in a CD4-BS dependent manner (Fig. 1C, open bars). Our second heterologous boost with the SOSIP cocktail, however, did not result in an apparent increase in the plasma antibody responses to these proteins (Fig. 1B, post week 18), including the CD4-BS fraction (Fig. 1C, filled bars).

### Characterization of Env+ B-cell receptors isolated after the final immunization

Two weeks post final immunization, class-switched memory IgG+ Env + B cells were isolated from the spleens of the immunized animals and individually sorted. To isolate Abs capable of recognizing the VRC01 epitope within the trimeric HIV-1 Env spike, tetramers of 426c.TM.SOSIP (which lacks the Loop D N276 (S278A), and V5 N460 (T462A) and N463 (T465A) NLGSs (see Materials and Methods for additional information and [20]), along with eOD-GT8 and eOD-GT8.KO (D276N/W277F/R278T/D279A/D368R), to exclude non-CD4-BS B cells, were employed as baits during B-cell sorting. Of note, 426c.TM.SOSIP was not part of the immunogens in the SOSIP cocktail employed during the final boost. B cells that were 426c.TM.SOSIP+/eOD-GT8+/eOD-GT8.KO− were isolated and their HC/LC genes sequenced.

**Fig 2:**
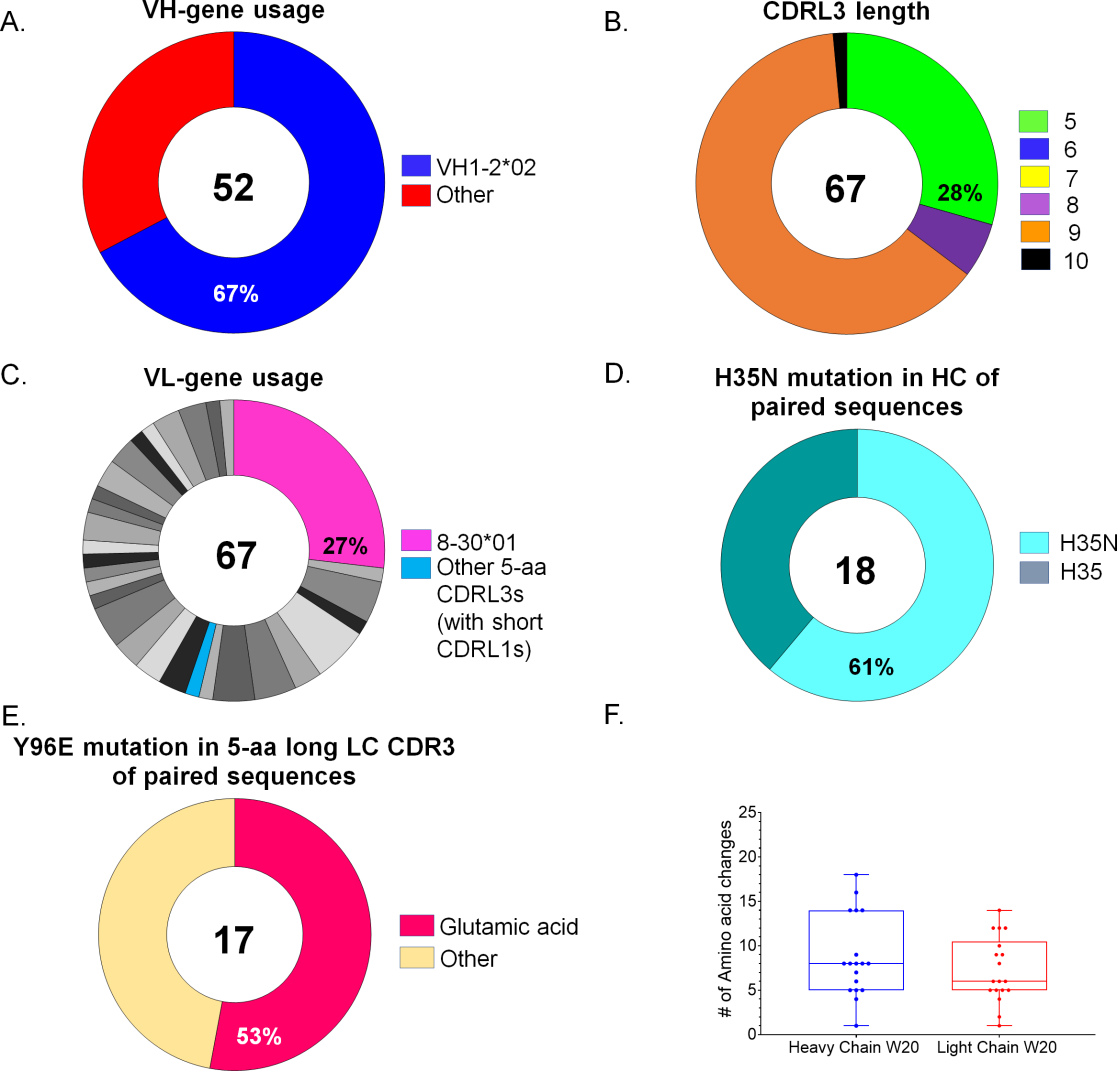
Heavy-chain/light-chain sequence analysis after the final boost immunization at week 20. Pie charts indicate HC (**A, D**) and LC (**B, C, E**) characteristics from individually sorted B cells from pooled mouse samples collected 2 weeks post final immunization. The number of HC and LC sequences analyzed is shown in the middle of each pie chart. (**A**) VH-gene usage, (**B**) aa length of the CDRL3 domains in the LC, and (**C**) LC-gene usage, where shades of gray/black slices represent non 5-aa-long CDRL3s. (**D–F**) Analysis of paired sequences: HCs with the H35N mutation are shown in (**D**), presence or absence of Glu_96_LC within the LC sequences with 5-aa-long CDRL3 domains are shown in (**E**), and (**F**) number of amino acid changes in the HC and LC of paired sequences at week 20, where each circle represents a paired sequence (see also Table S2).

A total of 162 Env+ class-switched memory B cells were sorted, and 52 HCs were successfully sequenced, of which 35 (67%) expressed the human inferred glVRC01 HC (Fig. 2A). Of the 67 LCs that were successfully sequenced, 19 (28%) contained the 5-aa long CDRL3s (Fig. 2B), consistent with our and other’s (24, 28, 31, 32) previous observations. The majority of LCs (95%, 18 of 19 5-aa-long CDRL3 containing sequences) were derived from the mouse κ8–30*01 LC V gene (Fig. 2C, shown in pink). The non-κ8–30*01 LC with a 5-aa-long CDRL3 was derived from the 12–46*01 V-gene (Fig. 2C, shown in blue). Interestingly, this LC was 9.7% somatically mutated and paired with VH1-2*02 HC.

In total, 61% (11 of 18) of the VH1-2*02 HC sequences that were paired with LCs expressing 5-aa-long CDRL3s contained an asparagine at position 35 instead of the gene-encoded histidine (H35N, in CDRH1, Fig. 2D). This H35N mutation introduces an additional hydrogen bond with N100a in CDRH3 and increases the stability of the interaction between CDRH1 and CDRH3 during the maturation of VRC01-class antibodies (28). In total, 53% (nine of 17) of the paired κ8–30*01-derived 5-aa-long CDRL3s contained a glutamic acid at position 96 (Glu_96,_ Fig. 2E), which is a key feature of mVRC01-class antibodies and forms a hydrogen bond with Gly459 in gp120 at the amino terminus of the V5 region (6, 7, 36).

The mean number of HC aa mutations in VH1-2*02 sequences that were paired with LCs expressing 5-aa-long CDRL3 was approximately 8.6, and the mean number of the corresponding LC aa mutations was approximately 7.3 (Fig. 2F). We previously reported that after the HxB2.WT.Core booster immunization, the mean number of aa mutations is approximately 6.6 and approximately 5.4 in the HCs and LCs, respectively (31). Thus, the immunization with the cocktail of SOSIPs increased the number of somatic mutations in VRC01-like BCRs. Importantly, despite the accumulation of somatic mutations, the three key amino acids, Trp_50HC_, Asn_58HC_, and Arg_71HC_, that make critical contacts with the CD4-BS of Env (7), remained unaltered, similar to what occurs during the affinity maturation process of VRC01-class bnAbs during HIV-1 infection (4, 5). Also, the fact that a range of mutations accumulated in the HCs and LCs of these BCRs (Table S2) indicates that B cells that express variations of the VRC01-like BCRs co-evolved in these animals following the SOSIP cocktail booster immunization.

### Env-binding properties of monoclonal VRC01-like antibodies isolated after the final immunization

**Fig 3:**
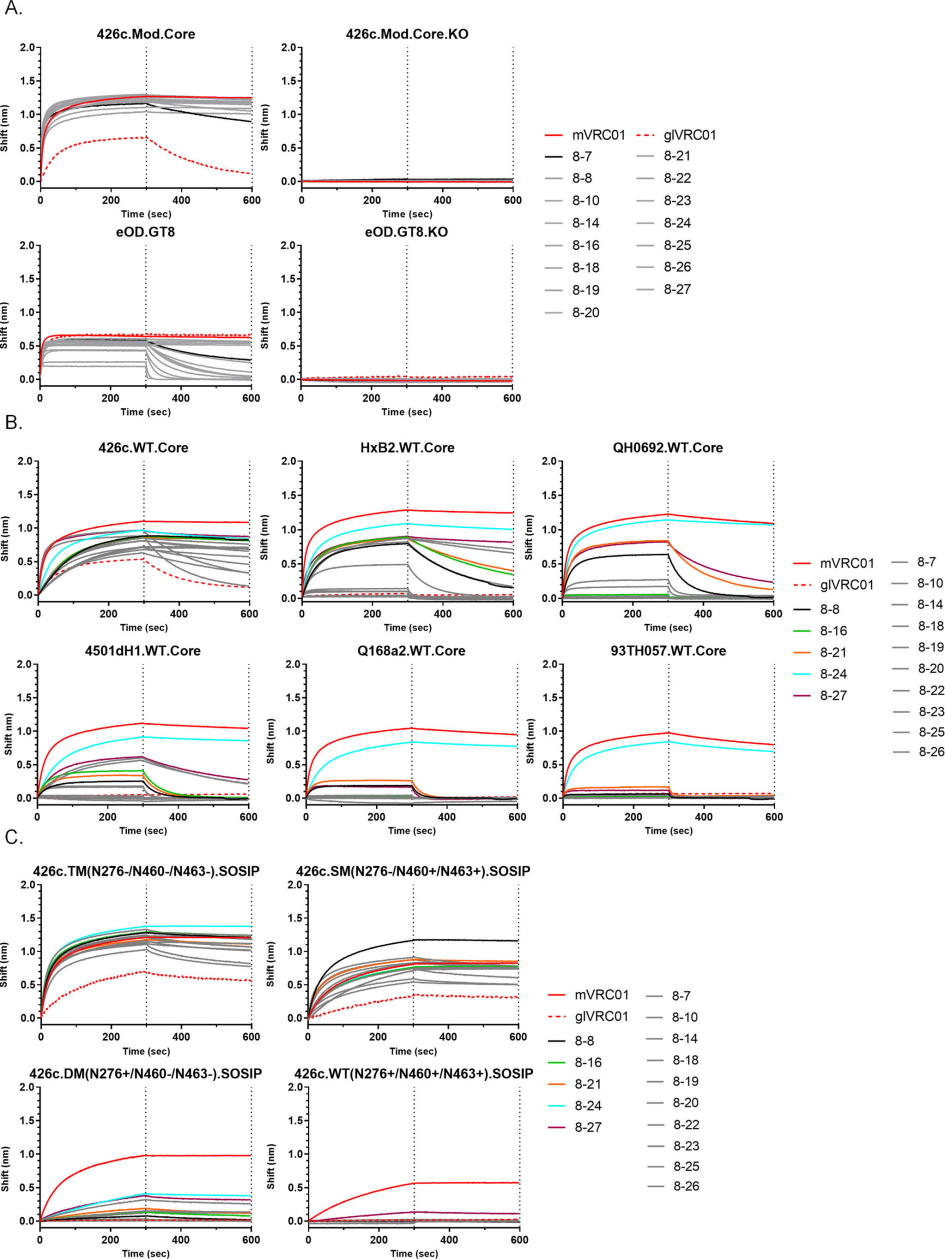
Binding properties of VRC01-like mAbs generated after the final boost immunization. (**A**) Fifteen VRC01-like mAbs were evaluated against the indicated soluble monomeric Envs. 8-7 mAb with fastest off rate for 426c.Mod.Core and eOD-GT8 is shown in black. (**B**) Binding of 15 mAbs against the indicated autologous and heterologous WT.Cores, where the mAbs that displayed cross-reactivity are shown in different colors. (**C**) Binding of 15 mAbs against the indicated variants of 426c.SOSIP are shown. mVRC01 (solid red line) and glVRC01 (dotted red line) were included as internal controls in all assays. Black dotted lines indicate end of association and dissociation phases (see also Tables S3 and S4).

To determine whether differences in aa mutations result in functional antibody differences, we generated IgGs from paired HC/LC sequences and investigated their binding and neutralizing properties. In total, we characterized 17 VRC01-like antibodies. Their VH and VL sequences are shown in Table S2, and their ontogenies are shown in Table S3. Sixteen antibodies expressed κ8–30*01-derived LCs with 5-aa-long CDRL3s, and one antibody (8-7) expressed the κ12–46*01 derived LC with 5-aa-long CDRL3s, as discussed above. Interestingly, mAb 8–27 with κ8–30*01-derived LC had a 7-aa-long deletion in its CDRL1 domain (Table S2). The Env-binding properties of these 17 VRC01-like antibodies were assessed using biolayer interferometry (BLI) (Fig. 3; Table S4).

We first confirmed that the antibodies recognize the CD4-BS by determining their binding to the 426c.Mod.Core Env and its CD4-BS KO version (Fig. 3A, top panel). The human mVRC01 and glVRC01 mAbs served as internal controls (Fig. 3, red, solid, and dotted lines). With the exception of 8–9 and 8–11 (data not shown), all mAbs bound 426c.Mod.Core, with 8–7 (in black) showing the fastest off-rate (Fig. 3A, top left panel), and as expected, none displayed any reactivity to 426c.Mod.Core.KO (Fig. 3A, top right panel). We next examined binding to eOD-GT8 (that only contains the outer domain of gp120 and lacks the N276 NLGS [16, 28]) and its CD4-BS KO version. All mAbs (including 8–9 and 8–11; data not shown) bound eOD-GT8 and not eOD-GT8.KO (Fig. 3A, bottom left and right panels, respectively). These results confirmed that the mAbs recognize the CD4-BS in the absence of glycans in Loop D (N276) and V5, but sequence variations affect their binding properties.

We next examined the binding of these VRC01-like mAbs to the autologous and various heterologous fully glycosylated gp120 cores (Fig. 3B). These proteins, described as WT.Cores, lack the V1, V2, and V3 domains (as well as the N and C termini of gp120), but are otherwise fully glycosylated, including at position N276 in Loop D and at the NXT/S sequon(s) in the V5 loop. A summary of the antibody recognition properties is shown in Table S4. All 15 mAbs bound the 426c.WT.Core with 8–7 and 8–26 showing the fastest off-rate, and 12 mAbs bound the heterologous HxB2.WT.Core, with mAbs 8–18, 8–19, and 8–26 not binding whereas mAbs 8–7, 8–20, and 8–23 showing very weak binding. Six of the 15 mAbs bound QH0692.WT.Core (clade B) with 8–24 displaying the slowest off-rate. Nine of the 15 mAbs bound 4501dH1.WT.Core (clade B) with 8–24 displaying the slowest off-rate. Four of the 15 mAbs bound Q168a2.WT.Core (clade A), of which only three bound the 93TH057.WT.Core (clade A/E), with 8–24 showing the strongest binding to all Envs (Fig. 3B). We conclude, therefore, that these VRC01-like antibodies can bypass the N276- and V5-associated glycans on autologous and heterologous gp120s lacking the variable domains 1–3, something that, as discussed above, the human glVRC01-class antibodies are unable to do (Fig. 3B, red dotted line). These results indicate that a heterogenous population of VRC01-like B cells co-exists in these immunized animals, with different CD4-BS recognition properties.

We next examined whether these antibodies could bind the VRC01 epitope on SOSIP proteins (i.e., in the presence of appropriately positioned V1–V3 domains) with and without NLGS at position N276 in Loop D and/or in V5 (Fig. 3C). All antibodies bound 426c.TM.SOSIP (N276-/N460-/N463-) (Fig. 3C, top left panel), indicating that these antibodies can bind in the presence of well-ordered V1–V3 loops when the Loop D and V5 NLGS are unoccupied. All antibodies also bound 426c.SM.SOSIP (N276-) (Fig. 3C, top right panel), indicating that they can bind in the presence of V1–V3 even when the V5 NLGS are present. While many mAbs were not able to bind to Env trimers that had the N276 glycan, mAbs 8–10, 8–21, 8–24, and 8–27 showed binding to a version of 426c.SOSIP that lacks the V5 NLGS (N460 and N463) but expresses the N276 NLGS in Loop D (426c.DM.SOSIP) (Fig. 3C, bottom left panel), with 8–10, 8–24, and 8–27 being the better binders. These data confirm that N276 poses the main obstacle for the maturing VRC01-like antibodies and also show that a subset of mAbs are able to partially overcome that obstacle. One antibody, 8–27, displayed binding (albeit very weak) to the fully glycosylated 426c.WT.SOSIP in this assay (Fig. 3C, bottom right panel). Collectively, these results indicate that most VRC01-like antibodies isolated at this stage of immunization can bypass the V1–V3 and N276 NLGS-associated glycans, as long as the V5 NLGS are unoccupied.

### mAbs 8–24 and 8–27 neutralize heterologous tier 2 viruses lacking N276-associated glycans

**Fig 4:**
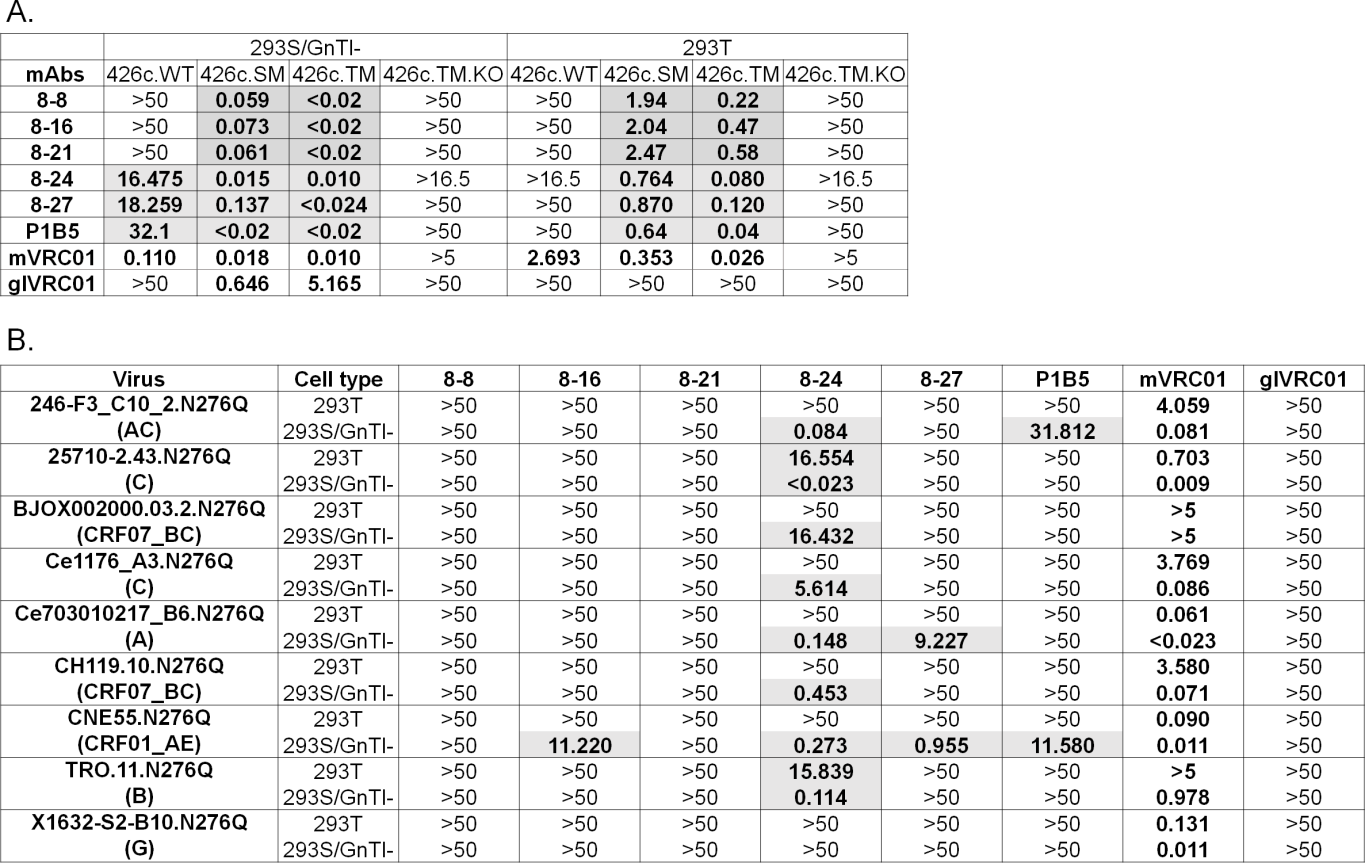
Neutralizing activities of VRC01-like mAbs. The neutralizing potential of mAbs against the indicated viruses either grown in 293T or 293S/GnTI− cells is shown in (A) and (B). Values represent IC50 neutralization in µg/mL. Bold/shaded values indicate samples displaying neutralizing activity. Neutralization IC50 values of these viruses with the mature VRC01 and germline VRC01 mAb are included for reference.

The neutralizing potentials of mAbs 8–8, 8–16, 8–21, 8–24, and 8–27, (which displayed the broadest cross-binding activities; Fig. 3 and Table S4) were determined against 426c.SM, 426c.TM, 426c.WT, and nine heterologous viruses with Envs lacking the N276 NLGS (N276Q virus) (Fig. 4). These viruses were produced either in 293T or 293S/GnTI− cells (i.e., expressing shorter glycans). With the exception of the 426c-derived viruses, which maintain their tier 2 phenotype when expressed in 293S/GnTI− cells (37), the other viruses become tier 1b when produced in 293S/GnTI− cells. All mAbs neutralized the 426c.SM and 426c.TM viruses produced in 293S/GnTI− cells more potently than glVRC01 (Fig. 4A). They also neutralized these two viruses when produced in 293T cells, while glVRC01 was unable to do so. The neutralizing activity of all antibodies was abrogated by the D279K mutation on the background of the 426c.TM virus (426c.TM.KO), confirming their VRC01 epitope recognition (37). Only mAbs 8–24 and 8–27 neutralized the 426c.WT virus when produced in 293S/GnTI− cells (but not in 293T cells), where glVRC01 did not neutralize either version of the 426c.WT virus. While the neutralizing potencies of these two antibodies against the 426c.SM and 426c.TM viruses grown in 293S/GnTI− cells were comparable to that of mVRC01 (except 8–27 against 426c.SM), their neutralizing potencies against the 426c.WT virus when produced in 293S/GnTI− cells was approximately 2 log_10_ lower than that of mVRC01.

Importantly, 8–24 neutralized eight of nine heterologous N276Q viruses when produced in 293S/GnTI− cells (Fig. 4B). Similar to glVRC01, mAbs 8–8 and 8–21 did not neutralize any of the tested viruses, while mAb 8–27 neutralized Ce703010217_B6.N276Q and CNE55.N276Q viruses produced in 293S/GnTI- cells, and mAb 8–16 only neutralized the CNE55.N276Q virus produced in 293S/GnTI- cells. This is possibly due to the 7-aa deletions in the CDRL1 of 8–27 or some improbable mutations (identified by ARMADiLLO (38)) that are unique to the heavy chain of mAbs 8–27 and 8–16 (Fig. S2). The 8–16 also shares some improbable mutations with mAb 8–27 HC (H35N) and 8–24 LC (QQYEY in CDRL3) that could be responsible for its observed neutralization pattern. Thus, antibodies 8–24 and 8–27 are more evolved than 8–8, 8–16, and 8–21, and bind the VRC01 epitope on functional Env spikes, when the overall length of the glycans around the CD4-BS are short. We do note, however, that 8–24 weakly neutralized TRO.11.N276Q and 25710–2.43.N276Q viruses even when produced in 293T cell (Fig. 4B).

We previously reported that antibodies with neutralizing activities against the 426c.SM and 426c.TM viruses expressed in 293S/GnTI− or 293T cells, and the 426c.WT virus expressed in 293S/GnTI− cells, and binding properties similar to those of 8–8, 8–16 and 8–21, can be isolated from these KI mice following the HxB2.WT.Core immunization (31). One such antibody is P1B5, whose neutralization potential is included in Fig. 4 for reference. However, P1B5 only neutralized two of nine N276Q viruses (less potently) and only when expressed in 293S/GnTI− cells. We conclude that the second boost immunization with the cocktail of SOSIPs improved the neutralizing properties of elicited VRC01-like antibodies. This improvement most likely is due to the selection of particular mutations by the SOSIP immunogens. It is also possible, however, that some of these mutations would have eventually emerged even in the absence of the SOSIP boost. Nevertheless, we have previously reported that extending the time between heterologous boost immunizations does not necessarily result in the development of antibodies with broader neutralizing activities (32).

### A short CDRL1 does not improve the neutralization potential of the partially mutated VRC01-like antibodies

**Fig 5:**
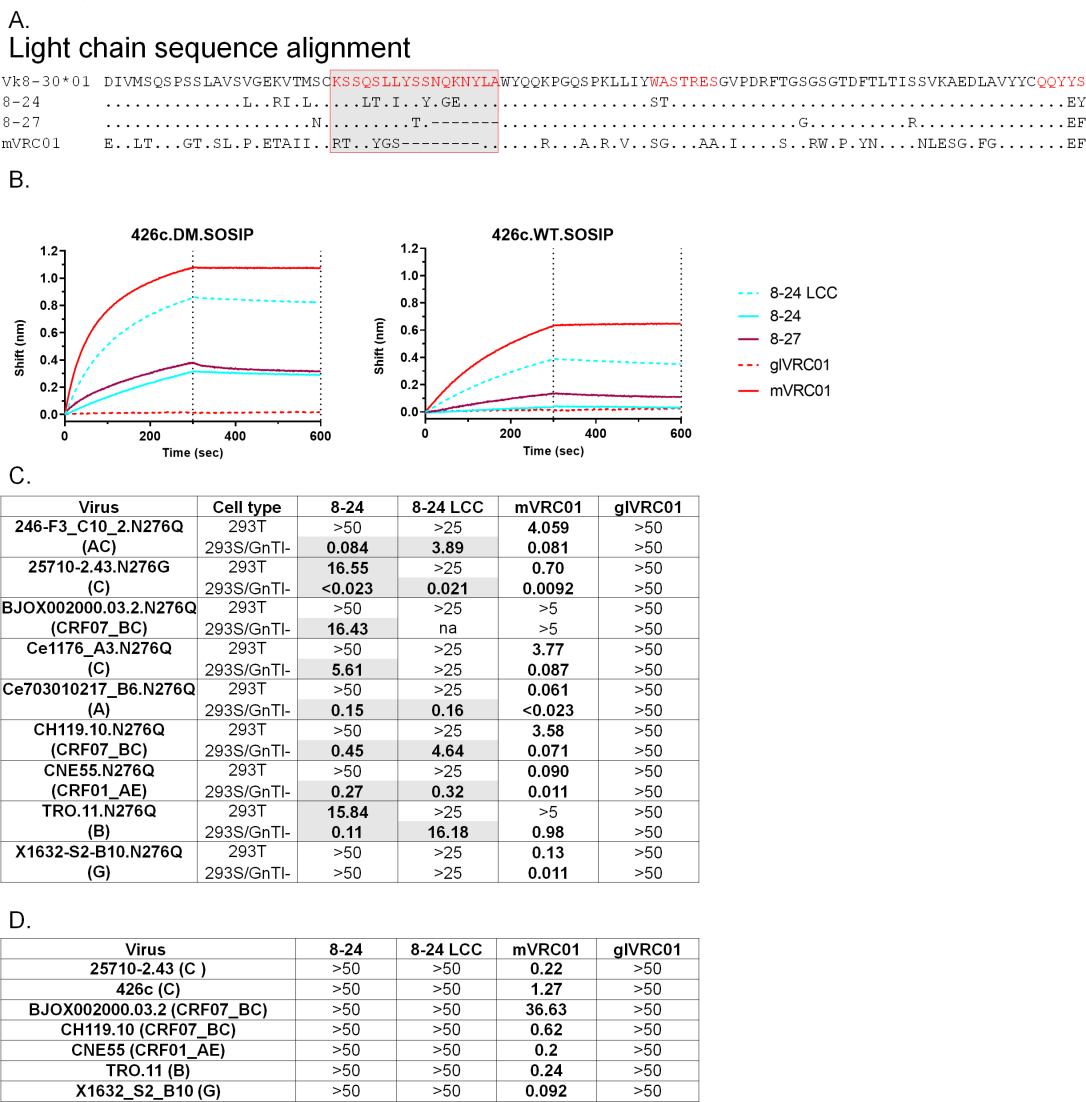
Binding and neutralizing properties of 8–24 chimeric mAb (8–24 LCC). (**A**) Sequence alignment of germline κ8–30*01 LC sequence, mAb 8–24, 8–27, and mVRC01 is shown. Red box/shaded region highlights the CDRL1 region; CDRL2 and CDRL3 residues on germline κ8–30*01 LC sequence are highlighted in red. (**B**) Binding of 8–24 LCC, 8–24, 8–27, mature, and germline VRC01 Abs against the indicated SOSIPs by BLI is shown. Black dotted lines indicate the end of association and dissociation phases. Neutralizing activities of 8–24 LCC against the indicated N276Q viruses either grown in 293T or 293S/GnTI- cells (**C**) or fully glycosylated WT viruses grown in 293T cells (**D**) is shown. IC50 neutralization values are provided in µg/mL, and the bold/shaded region indicates samples displaying neutralizing activity. Neutralization IC50 values of the same viruses with 824 mAb, mature VRC01, and germline VRC01 mAb are included for reference.

As discussed above, some human VRC01 mAbs accumulate CDRL1 indels (resulting in a shorter, 7- to 11-aa-long CDRL1, compared to 12 in glVRC01 and 17 in κ8–30*01) (6). These evolutionary changes are believed to be necessary for the antibody LCs to accommodate the N276-associated glycans on full-length trimeric Envs. The 8–24 has not acquired such indels (Fig. 5A). In contrast, the CDRL1 of 8–27 was reduced by 7 aa (i.e., 10-aa long), and as a result, it was closer in length to that of the mature human VRC01 mAb (which is 9-aa long) (6). The above-discussed neutralization results imply that the shorter CDRL1 expressed by 8–27 (10 vs 17 aa) may contribute to its broader neutralizing potential compared to those of 8–8, 8–16, and 8–21, but does not explain why it has narrower neutralizing activity than 8–24 whose CDRL1 is 17-aa long. To address this critical point, we replaced the CDRL1 of 8–24 with the shorter CDRL1 of the human mVRC01 and generated the chimeric mAb (8–24 LCC). We then tested the binding capacity of 8–24 LCC to 426c.WT.SOSIP and 426c.DM.SOSIP. Indeed, we observed increased binding against both Envs in the chimeric mAb (Fig. 5B, cyan dotted line). The neutralizing activity of 8–24 LCC was then compared to that of 8–24 against heterologous N276Q viruses expressed in 293T and 293S/GnTI− cells (Fig. 5C). However, CDRL1 shortening resulted in similar (or weaker in some cases) neutralizing activity of mAb 8–24 LCC against these viruses (Fig. 5C). This suggests that CDRL1 length by itself does not regulate the neutralizing potential of VRC01-class antibodies, but rather cooperates with other domains of these antibodies that also accumulate mutations over time. Similarly, the neutralizing activity against the autologous 426c and six other heterologous viruses expressing full-length glycans was not improved for 8–24-LCC (Fig. 5D). The 8–27 mAb did not neutralize any of these viruses expressing full-length glycans either (data not shown). We therefore conclude that a shorter CDRL1 does not improve the neutralizing abilities of antibodies like 8–24, even though it increased the binding in BLI assay (Fig. 5B and D).

### Cryo-EM structure of mAb 8-24 bound to a soluble Env trimer

**Fig 6:**
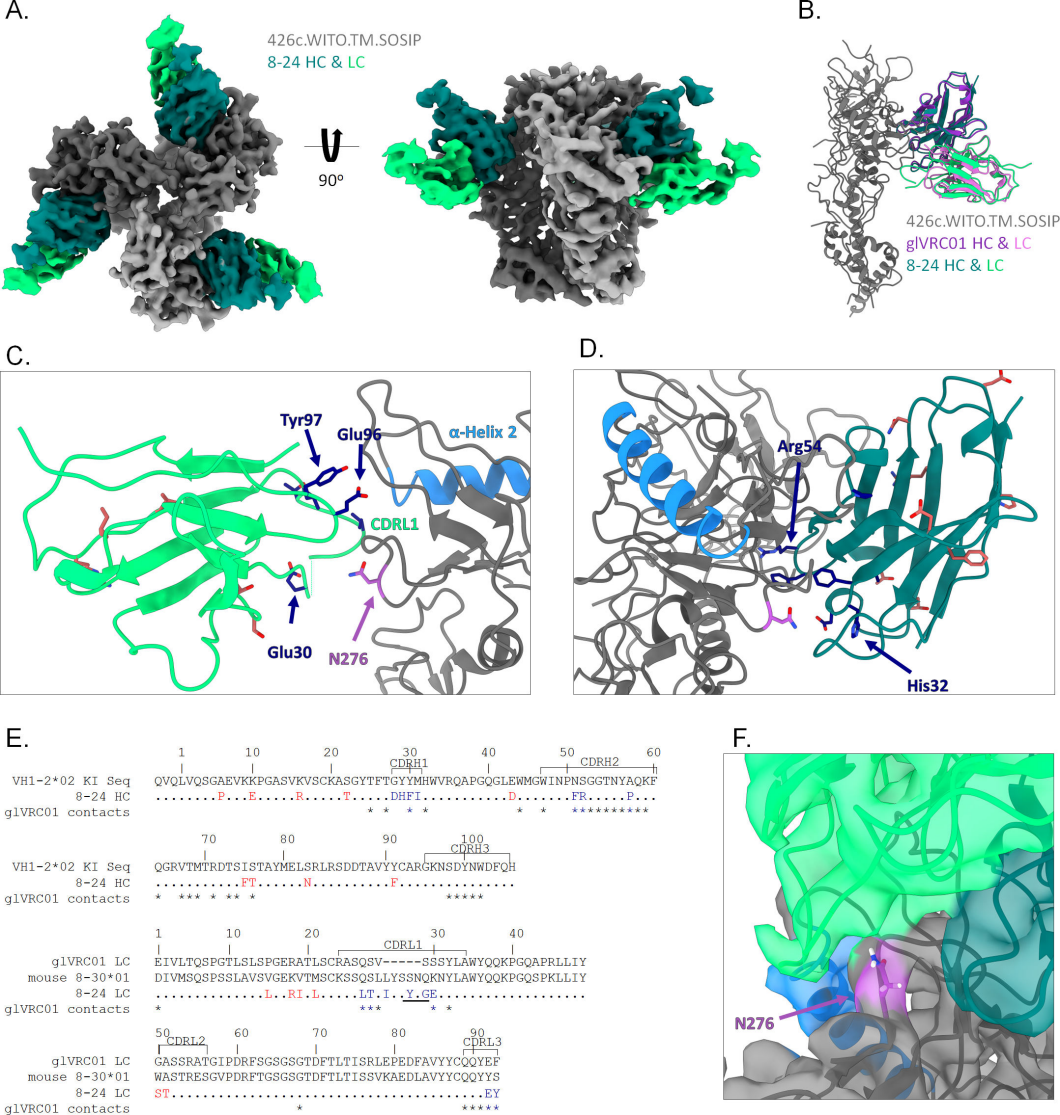
Cryo-EM structure of mAb 8–24 bound to 426c.WITO.TM.SOSIP. (**A**) Cryo-EM map of 426c.WITO.TM.SOSIP with 8–24 Fab shown in two orientations. SOSIP protomers are colored in shades of gray, HC of mAb 8–24 is shown in teal, and LC in light green. (**B**) Alignment of mAb 8–24 and glVRC01 (from PDB ID 6MYY) on a protomer of 426c.WITO.TM.SOSIP shows nearly identical angle of approach and epitope. (**C**) View of mAb 8–24 LC highlighting matured residues potentially involved in binding (dark blue) and matured residues in the framework regions (red). The CDRL1 comes in close proximity to the gp120 α-helix 2 (shown in blue). N276 is shown in purple. (**D**) View of mAb 8–24 HC highlighting matured residues potentially involved in binding (dark blue) and matured residues in the framework regions (red). gp120 α-helix 2 is shown in blue. N276 is shown in purple. (**E**) Sequence alignments of mAb 8–24 HC with human VH1-2*02 KI sequence, and mAb 8–24 LC with glVRC01 LC and mouse 8–30*01 LC. For mAb 8–24, affinity matured residues are colored to reflect if they are potentially involved in binding (dark blue) or not involved (red). Residues of glVRC01 that are involved in binding to 426c.WITO.TM.SOSIP are indicated by stars. Residues not resolved in the CDRL1 of mAb 8–24 are underlined. (**F**) mAb 8–24 bound to 426c.WITO.TM.SOSIP model and cryo-EM map show a narrow channel that could accommodate short glycans at the N276 position.

To better understand why 8–24 neutralizes the autologous tier 2 426c.WT virus when expressed in 293S/GnTI- cells but not when expressed in 293T cells (Fig. 4A), we carried out structural analysis using cryo-EM. Due to the weak binding of 8–24 to 426c.WT.SOSIP (Fig. 3C, bottom right panel), we were unable to generate a complex of 8–24 antigen-binding fragment (Fab) bound to 426c.WT.SOSIP. However, we obtained a cryo-EM reconstruction to 4.2-Å resolution of 8–24 bound to a variant of 426c.TM.SOSIP whose V1/V2 loops have been substituted by those of the heterologous WITO Env (426c.WITO.TM.SOSIP) (REF PMID: 26689967) (Fig. 6A; Fig. S4; Table S5). We screened numerous variants of the 426c.TM.SOSIP by cryo-EM, where 426c.WITO.TM.SOSIP produced the most homogenous particles and highest resolution reconstruction. To assist in model building of the cryo-EM structure, a crystal structure of unbound mAb 8–24 was obtained at a resolution of 2.01 Å (Fig. S3; Table S6), in which the long CDRL1 is disordered.

In the complex, the SOSIP is in the closed, prefusion conformation (Fig. 6A). Both mVRC01 and glVRC01 have been shown to bind to the closed Env trimer as well (39). An alignment with a structure of glVRC01 bound to 426c.TM.SOSIP (referred to as degly-3 in PDB ID 6MYY, (39)) showed that mAb 8–24 approaches the VRC01 epitope with the same angle of approach as glVRC01, with the two structures having an overall RMSD of 0.72 Å over 632 alpha carbons (Fig. 6B). Due to the low resolution of the structure, we could not definitively model in the side chains for a more in-depth structural analysis of the interactions and used comparison with other known structures (PDB ID 6MYY, [39]). Overall, the main contacts made by glVRC01 that are gl-encoded were maintained in mAb 8–24: Thr28, Thr30, Trp47, Trp50, Gly55, Gly56, Asp58, Tyr59, Gln61, Lys62, Gln64, Val67, Thr68, Met69, Arg71, Thr73, and Ser74 in the HC; and Tyr32, Gly68, Gln89, Gln90, Tyr91, and Glu96 in the LC (following Kabat numbering). We highlighted the residues in the light and heavy chains of mAb 8–24 that are somatically mutated from germline and distinguished those involved in binding to 426c.TM.SOSIP (Fig. 6C through E). Both the CDRH1 and CDRL3 have numerous mutations that could interact with the CD4-BS, leading to an increase in binding and neutralization. Notably, there are many mutations in the framework regions. While these do not directly interact with the SOSIP, framework region mutations have been shown to increase the breadth and potency of neutralization in HIV-1 antibodies (40). Moreover, the long CDRL1 of 8–24 appears to come into proximity to the C-terminal end of gp120 α-helix 2, potentially creating an interaction that is lacking in VRC01-class antibodies with a short CDRL1 (Fig. 6C). This long CDRL1 reaches toward the N276 glycan and, along with the CDRH3, appears to create a channel that could accommodate the shorter glycans at the N276 site (Fig. 6F), suggesting that the interaction between the long CDRL1 and the α-helix 2 may be possible if the glycans expressed on N276 are short, as is in the case of Envs produced in 293S/GnTI- cells. This could explain why 8–24 neutralizes the 426c.WT virus when expressed in GnTI- cells, but not when expressed in regular 293T cells (Fig. 4A).

## DISCUSSION

Here, we examined whether amino acid deletions (indels) in the CDRL1 of VRC01- like antibodies are necessary for their broadly neutralizing activities. Such deletions are present in several, but not every, VRC01-class bnAb that has been isolated from people with HIV-1 (1, 4–6, 41), and they are believed to be a key step in the maturation process of VRC01-class antibodies, which leads to the acquisition of neutralizing breadth. So far, however, CDRL1 indels are very rarely elicited during immunization of KI mice expressing human VRC01-class BCRs (25, 26).

To examine whether the absence of CDRL1 indels is indeed the reason for the lack of neutralizing breadth of vaccine-elicited VRC01-like antibodies in KI mice, we employed the VRC01^glHC^ KI mouse model (24, 28, 31). In this model, vaccine-elicited VRC01-like antibodies express the full-length inferred germline HC of the human VRC01 mAb, paired with mouse κ8–30*01 LCs, that have 17-aa-long CDRL1.

We previously reported that a priming immunization with the gl-targeting clade C 426c.Mod.Core nanoparticle and booster immunization with the heterologous clade B HxB2.WT.Core nanoparticle immunogens result in the activation and partial maturation of VRC01-like antibodies in the above KI mouse model (31, 32). VRC01-like antibodies elicited after the HxB2.WT.Core immunization can efficiently neutralize viruses expressing Env lacking the N276 NLGS, but fail to neutralize the corresponding viruses expressing the N276 NLGS (31, 32). Here, we immunized the mice a third time with a cocktail of soluble stabilized heterologous SOSIP Envs from different clades (B/C/M/H), to select for antibodies with somatic mutations that would allow them to engage the VRC01 epitope on functional, trimeric Envs. This third immunization led to the accumulation of additional mutations compared to our previously published results after two immunizations (31, 32). B cells can acquire somatic mutations over time independent of repetitive boosting, but based on our previously published (32) results, we conclude that an additional immunization with a SOSIP cocktail resulted in the observed boosting effect in SHM rates of the isolated mAbs. While the majority of the elicited VRC01-like antibodies did not express CDRL1 indels, one antibody (8–27) had a 7-aa CDRL1 deletion. Surprisingly, mAb 8–27 displayed similar neutralizing potential to VRC01-like antibodies, which did not express CDRL1 deletions. To confirm a role for short CDRL1, we therefore replaced the long CDRL1 (17 aa in length) of 8–24 (mAb showing broadest cross-binding activity in the study) by the short one (9 aa in length) present in the human VRC01-class bnAb VRC01 (6). The chimeric antibody (8–24 LLC) displayed improved binding to 426c.WT.SOSIP but weaker neutralizing breadth compared to the parental mAb. Therefore, our results strongly suggest that CDRL1 deletions in the mutated VRC01-like antibodies generated in this KI mouse model may not be sufficient for the development of neutralizing breadth.

We would like to highlight the fact that similar observations were made in a different KI mouse model immunized with a different set of Env immunogens (25). This mouse expresses gl-CH31 BCRs and, when administered with SOSIP cocktails at the end of the prime-boost immunization schema, elicits VRC01-like Abs with two amino acid CDRL1 deletions that do not display broad neutralizing activities. Similarly, in a third KI mouse (expressing gl-VH1-2*02 paired with the gl κ3–20*01 LC) immunized with a series of nine immunogens, vaccine-elicited VRC01-class antibodies with two to three amino acid CDRL1 deletions did not display better neutralizing breadth than VRC01-class antibodies without CDRL1 deletions (26). Thus, we do not believe that the results presented here are unique to the KI mouse model we used. Barnes et al. reported that CDRL1 deletion on the broadly neutralizing BG24 VRC01-class antibody, which was isolated from an HIV + individual, was important for maintaining the neutralizing potential of that antibody (1). Although deletions in CDRL1, or glycine substitutions in CDRL1, improve the binding of the maturing VRC01-class antibodies to Env, they do not always result in improvements in neutralizing potency ([1, 6, 9, 25, 26, 35, 36, 42], and data presented here in Fig. 5), and so far, there is no evidence that they improve the neutralizing breadth of these antibodies.

Overall, we propose that mutations in CDRL1 alone will be insufficient to confer broad neutralizing properties to the maturing VRC01-class antibodies and that a well-orchestrated accumulation of specific types of somatic mutations outside of CDRL1 may lead to the development of VRC01-class antibody neutralizing breadth by immunizations. For instance, longer CDRL1s may be more flexible than short ones and therefore accommodate the N276-associated glycans more efficiently. However, since additional steric blocks are imposed by the lengths of the V1, V2, and V3 domains (19), longer CDRL1s will also have to accommodate those regions. Knock-in mouse models expressing VRC01-class BCRs with different CDRL1 lengths can be used to examine whether and how BCRs, which differ only in the lengths of their CDRL1, can mature into broadly neutralizing antibodies or not. Additionally, certain SOSIPs in the cocktail used here may be more helpful than others in selecting the right mutations because, as mentioned above, the binding of germline or partially mutated VRC01-class antibodies to Env depends not only on the Loop D and V5 glycans but also on the length of the V1, V2, and V3 regions. Thus, in the future, selection of the most appropriate SOSIPs may lead to the elicitation of antibodies that display neutralizing activities against viruses that express full-length Envs.

## MATERIALS AND METHODS

### Recombinant HIV-1 envelope proteins and tetramers

Recombinant HIV-1 Env proteins were expressed by transient transfection in HEK 293F cells and then purified directly from conditioned media as we previously described (19). The 426c.Mod.Core is derived from the gp120 of the clade C 426c Env. It has deletions in the V1/V2/V3 and lacks the N276 (loop D) and N460 and N463 (V5) NLGS, along with the G471S mutations (19). The “CD4-BS knockout” version of 426c.Mod.Core contains the D279A, D368R, and E370A mutations. The HxB2.WT.Core is derived from the gp120 of the clade B HxB2 Env with deletions in V1/V2/V3 (31). The KO version of eOD-GT8 contains the D368R mutation, and the amino acids DWRD at positions 276–279 were substituted by NFTA. Purified Env proteins were aliquoted in phosphate-buffered saline (PBS) and stored frozen in −20°C until further use. Self-assembling nanoparticles expressing 426c.Mod.Core and HxB2.WT.Core were produced and purified as previously described (19). They were stored at 4°C. SOSIP proteins (Table S1) were produced as previously described. Tetramers of Avi-tagged eOD-GT8, eOD-GT8.KO, and 426c.TM.SOSIP (TM: S278A/T462A/T465A) were generated as previously reported (19, 30–32).

### Mice, immunizations, and sample collection/processing

Knock-in mice expressing the inferred germline HC of the human VRC01 Ab (VRC01^glHC^) and endogenous mouse LCs (28) were bred and kept at the animal facility in Fred Hutchinson Cancer Center. Mice were 6- to 12-week old at the initiation of experiments. Proteins and adjuvants were diluted in PBS and administered intramuscularly with 50 µL in each hind leg in the gastrocnemius muscle (total volume 100 µL/mouse). Env antigens were administered at 50 µg/mice (426c.Mod.Core and HxB2.WT.Core Ferritin nanoparticles), or 10 µg/mice (SOSIP cocktail), and GLA-LSQ at 50 µg for 426c.Mod.Core and HxB2.WT.Core, and 10 µg for SOSIP cocktail. Blood was collected by the retro-orbital route into tubes containing 25 µL of citrate–phosphate–dextrose solution (Sigma-Aldrich). Terminal bleeds were collected by cardiac puncture into tubes containing 100 µL of citrate phosphate–dextrose solution. Plasma was isolated from blood, heat inactivated at 56°C, and stored short term at 4°C for further analysis. Organs were harvested into cold IMDM media (Gibco), and organ processing for spleens and lymph nodes was carried out as previously described (32).

### ELISA

A total of 384-well ELISA plates (Thermo Fisher Scientific) were coated with 0.1 µM his/avi-tagged protein (426c.Mod.Core, 426c.Mod.Core.KO, HXB2.WT.Core, HXB2.WT.Core.KO, eOD-GT8, and eOD-GT8.KO) diluted in 0.1 M sodium bicarbonate, at room temperature (RT) overnight. Plates were then washed four times with wash buffer (PBS plus 0.02% Tween20) using a microplate washer (BioTek) and incubated with block buffer (10% milk, 0.03% Tween20 in PBS) for 1–2 h at 37°C. Plates were washed, mouse plasma added, and serially diluted (1:3) in block buffer. After 1 h of incubation at 37°C, plates were washed, and horse radish peroxidase-conjugated goat anti-mouse IgG (BioLegend) was added and incubated for 1 h at 37°C. After a final wash, SureBlue Reserve TMB Microwell Peroxidase Substrate (KPL Inc.) was added to the plates for 5 min. The reaction was stopped with 1 NH_2_SO_4_, and the optical density (OD) was read at 450 nm with a SpectraMax M2 Microplate reader (Molecular Devices). The average OD of blank wells from the same plate were subtracted from all wells before analysis using Prism software.

### Single B-cell sorting and HC/LC gene sequencing

Splenocytes or lymph node cells were thawed and stained as previously described (32). In total, 1 µM of eOD-GT8, eOD-GT8.KO, and 426c.TM.SOSIP tetramers was used as baits. Samples were single cell-sorted into 96-well skirted plates (Eppendorf) containing lysis buffer (previously described) and stored at −80°C until further processing. Amplification and sequencing of the antibody HC/LC genes was performed as we previously described (30–32). HC and LC sequences were analyzed using the Geneious software (Biomatters, Ltd.) and the online IMGT/V-QUEST tool (30–32). To calculate the number of amino acid mutations, sequenced HC and LC pairs were aligned against the VH regions of the VRC01 glHC knocked-in sequence and LC reference sequences obtained from IMGT/V-QUEST, respectively, using sequences starting from CDR1 to CDR3. HC/LC sequences are provided in Table S2. The ARMADiLLO web server (https://armadillo.dhvi.duke.edu) was used to analyze antibody sequences and display the probability estimates for all possible amino acid changes over the full length of an antibody sequence.

### HC/LC cloning and antibody expression

DNA products from the first round of PCR were used as templates for gene-specific PCR to amplify the gene of interest, and ligation sites were added to allow for insertion of the DNA fragment into the human IgG1 vectors: ptt3 for κ light chain (43) and PMN 4–341 for γ heavy chain (44). PCR reactions were performed as previously described (32). The gene-specific PCR product was then infused into cut IgG1 vector in a 2.5-µl volume reaction containing 12.5 ng of cut vector, 50 ng of insert, and 0.5 µL of Infusion enzyme (Takara Bio). To generate the 8–24 chimeric antibody (8–24 LCC) with mVRC01 CDRL1, two primers flanking the CDRL1 of 8–24 were designed such that one primer contained the VRC01 CDRL1-base gene segments, and the other served to linearize the 8–24 LC plasmid. PCR was performed with 1 ng of template and 0.5 µM of forward and reverse primers using the Platinum SuperFi II polymerase master mix (Thermo Fisher), and the PCR product was cloned into the linearized 8–24 LC vector using the In-Fusion HD Cloning Plus system (Takara Bio). Competent *E. coli* cells were transformed with the entire reactions and plated onto ampicillin agar plates. Colonies were picked and grown in LB broth containing ampicillin, and DNA was extracted and purified using QIAprep Spin Miniprep Kit (Qiagen). The 293E cells were then transfected with equal amounts of HC and LC DNA as well as 293F transfection reagent (Millipore Sigma) or PEI and grown for 5–7 days, at which time Abs were purified from cell supernatants using Pierce Protein A agarose beads (Thermo Fisher Scientific). Abs were eluted with 0.1 M citric acid into 1 M Tris buffer followed by buffer exchange into PBS using an Amicon centrifugal filter (Millipore Sigma).

### Biolayer interferometry

BLI assays were performed on the Octet Red instrument (ForteBio) as previously described (19, 30–32). Briefly, anti-human IgG Fc capture biosensors (ForteBio/Sartorius) were used to immobilize mAbs (20 µg/µL in PBS) for 5 min, followed by baseline interference reading for 60 s in kinetics buffer (PBS, 0.01% bovine serum albumin, 0.02% Tween-20, 0.005% NaN_3_). Sensors were then immersed into wells containing different Envs (2 µM) diluted in kinetics buffer for 300 s (association phase) and another 300 s (dissociation phase). mVRC01 and glVRC01 mAbs were used as internal controls for comparison. All measurements were corrected by subtracting the signal obtained from simultaneous tracing of the corresponding Env using an irrelevant IgG Ab in place of the mAbs tested. Curve fitting was performed using the Data analysis software (ForteBio).

### TZM-bl neutralization assay

Generated mAbs were tested for neutralization against a panel of selected HIV-1 pseudoviruses using TZM-bl target cells, as previously described (45). Germline and mature VRC01 mAbs were used as references in every assay.

### Protein expression and purification for structural studies

The variable region of the HC of mAb 8–24 was cloned into a pMN vector with the human CH1 domain and a C-terminal His-tag to directly express the Fab using the In-Fusion HD Plus cloning system (Takara Bio). mAb 8–24 LC was also cloned into a pMN vector to improve expression of the mAb 8–24 Fab. PCR reactions and cloning were performed as described above (HC/LC cloning and antibody expression section). mAb 8–24 Fab was expressed through transient transfection in 293E cells using the Freestyle 293 expression system and PEI as a transfection reagent for 6 days at 37°C and 6% CO_2_. mAb 8–24 Fab was purified using Ni-NTA affinity resin followed by size exclusion chromatography (SEC) on a Superdex200 16/600 column (Cytiva). The 426c.WITO.TM.SOSIP, a 426c.TM.SOSIP where the V1/V2 loops are replaced by the V1/V2 loop of the WITO Env (46), was expressed in 293F/GnTI− cells using the Freestyle 293 expression system and co-transfected with furin to facilitate cleavage of the gp120 and gp41. The SOSIP was purified from cultural supernatant 6 days after transfection using a mVRC01 IgG affinity column and SEC on Superdex200 10/300 column (Cytiva).

### Crystallization of mAb 8-24

Crystallization conditions were screened at RT using the sitting drop vapor diffusion method with a Formulatrix NT8 drop setting and Rock Imager. Screening was done with the MCSG1-3 screens (Microlytic) and a Fab concentration of 15 mg/mL. Crystals from the MCSG1 F6 condition (20% PEG3350 and 0.2 M KSCN) were further optimized using the hanging drop method at RT. Final crystals were grown in 30% PEG3350 and 0.4 M KSCN. A cryoprotectant of 30% PEG3350, 0.4 M KSCN, and 30% ethylene glycol was used. Crystals diffracted to 2.01 Å. Data were collected at Advanced Light Source on beamline 5.0.2, processed with XDS (47), and scaled using AIMLESS in CCP4 (48). The structure of mAb 8–24 His Fab was solved by molecular replacement using glVRC01 of 7JLN (chains H and L) as a search model Phaser in Phenix (49). Structural refinement was performed using phenix.refine and COOT (50). The mAb 8–24 Fab structure has two Fabs in the asymmetric unit. Coordinates were deposited in the PDB under 9B44. Figures were made in ChimeraX (51).

### cryoEM complex and grid preparation

A 3.5 M excess mAb 8–24 Fab was mixed with 426c.WITO.TM.SOSIP, incubated at 4°C for 1 h, and then purified on Superose6 10/300 column. Fractions corresponding to the complex were pooled and concentrated. Initial cryo-EM screening showed almost all SOSIP trimers falling apart into monomers. To limit this SOSIP breaking, prior to grid freezing, the complex was treated with glutaraldehyde at a concentration of 0.25% for 45 s followed by quenching in 1 M Tris, pH 7.5. Complex was buffer exchanged and concentrated in 1× TBS. A Vitrobot (MARKIV) was used to prepare cryo-EM grids. Dodecyl-β-D-maltoside was added to the sample at 0.05% final concentration 20 min prior to freezing. Three microliters of the complex at 1.5 mg/mL was loaded on UltrAuFoil R2/2 grids on a 200 mesh (Ted Pella). Grids were blotted for 6 s with a blot force of 5 at 22°C and 100% humidity before plunging into liquid ethane

### cryoEM data collection and analysis

Data were collected on a Glacios microscope at 200 kV equipped with a K3 direct electron detector. Initial screening showed orientation bias, and CryoEF (52) was used on COSMIC2 (53) to determine the tilt for further data collection. Data were collected in 50 frame movies using Serial EM (54) at ×36,000 (1.122 Å/px), and 3,438 movies were collected at a 28° tilt. Motion correction was performed using WARP (55). Motion-corrected micrographs were loaded into cryoSPARC v4.2 (56).

A blob picker was used to pick initial particles. From a subset of 50 micrographs, 12,182 particles were 2D classified, and 15 classes with 3-Fabs bound were used for template picking. From template picker, 2.6 million particles were selected and extracted with a box size of 384 px. Following three rounds of 2D classification (100 classes, 100 classes, and 50 classes), 95,299 particles with three Fabs bound remained. A single *ab initio* model was generated and refined. A 3D classification was used to remove low-resolution particles, and homogeneous refinement with C3 symmetry of the 3D classes showed much higher resolution for one class (4.6 vs approximately 7.5 Å). This higher resolution class of 23,004 particles was locally refined to 4.25-Å resolution.

The cryoEM structure of 426c.WITO.TM.SOSIP bound to glVRC01 (PDB ID 6MYY) was docked into the sharpened map for the SOSIP, and the crystal structure of mAb 8–24 Fab was aligned to glVRC01. Further model building was done using COOT (50) and ISOLDE (57) in ChimeraX (51). Structural refinement was done using Phenix (49). Figures were generated using ChimeraX. Model and maps were deposited to the PDB (9BGE) and EMDB (44510).

## Data Availability

Binding and neutralization results and data collection and refinement statistics for cryo-EM structure related to Fig. 6 are available under PDB ID 9BGE and 9B44.

## SUPPLEMENTAL MATERIAL

Figure S1 jvi.00744-24-s0001.tif Plasma antibody responses.

Figure S2 jvi.00744-24-s0002.tif Estimated probabilities of observed mutations in mAbs 8-16 and 8-27.

Figure S3 jvi.00744-24-s0003.tif Crystal structure of mAb 8-24.

Figure S4 jvi.00744-24-s0004.tif Cryo-EM map processing and resolution pipeline.

Supplemental material jvi.00744-24-s0005.docx Tables S1, S3, S5, and S6; Figure legends.

Table S2 jvi.00744-24-s0006.xlsx HC/LC sequences of the VRC01-like antibodies.

Table S4 jvi.00744-24-s0007.xlsx Summary of overall binding properties of the 15 VRC01-like mAbs.

ASM does not own the copyrights to Supplemental Material that may be linked to, or accessed through, an article. The authors have granted ASM a non-exclusive, world-wide license to publish the Supplemental Material files. Please contact the corresponding author directly for reuse.
