# Supplementary figures and images for: Short CDRL1 in intermediate VRC01-like mAbs is not sufficient to overcome key glycan barriers on HIV-1 Env
Source: J Virol. 2024 Sep 6;98(10):e00744-24. doi: 10.1128/jvi.00744-24 (PMC11495006; doi:10.1128/jvi.00744-24)

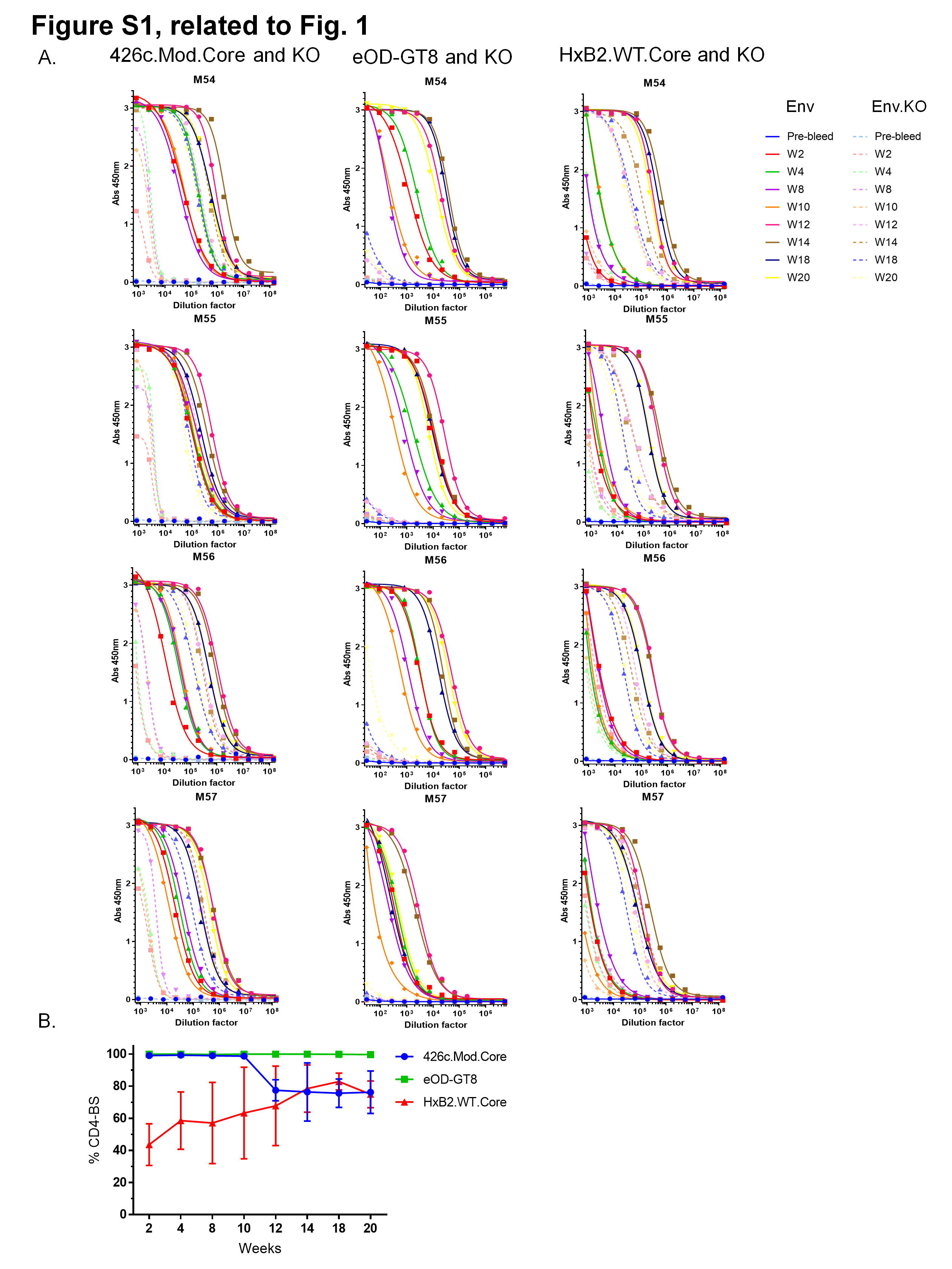

Supplement: Figure S1 — Plasma antibody responses. [file jvi.00744-24-s0001.tif]

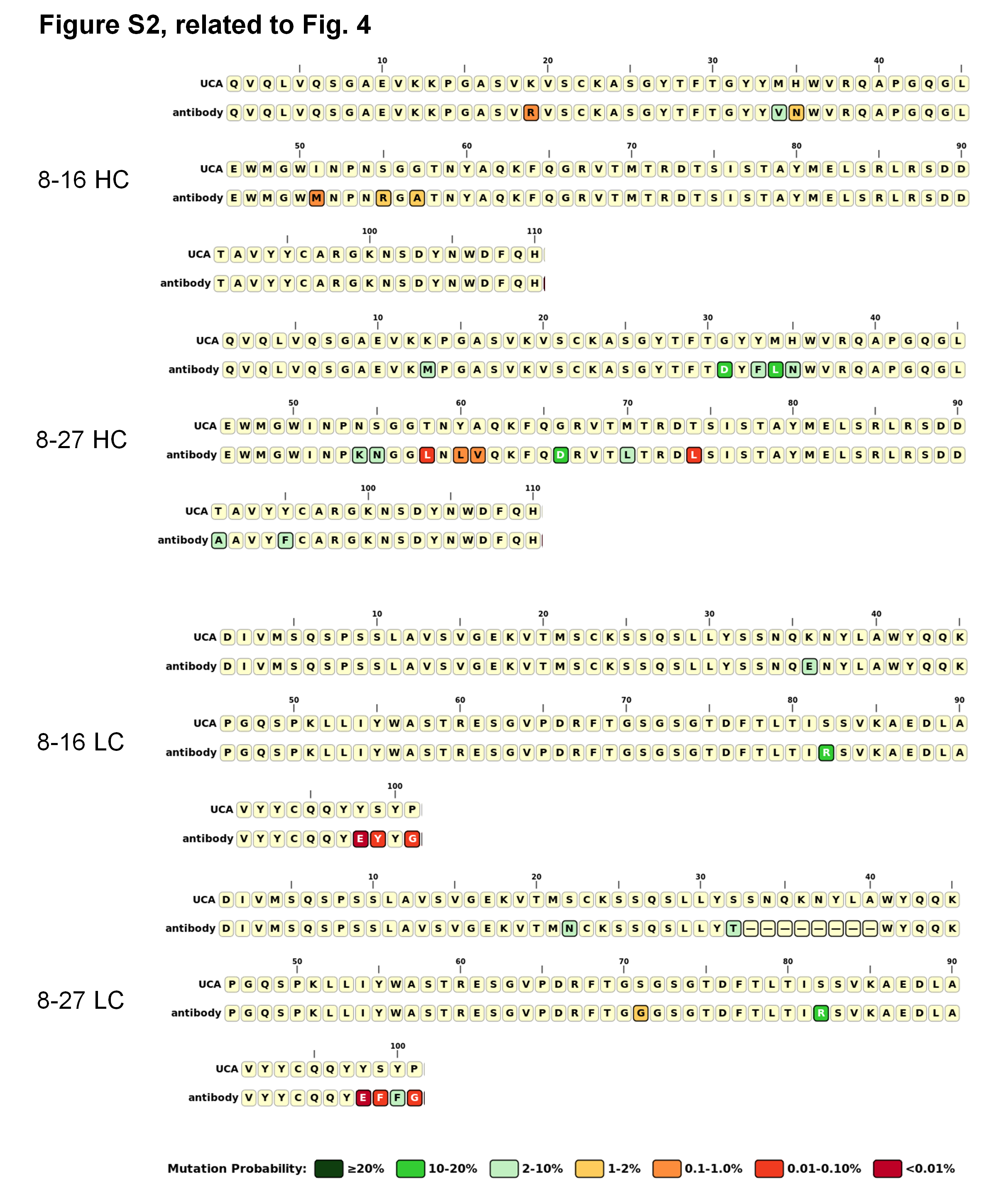

Supplement: Figure S2 — Estimated probabilities of observed mutations in mAbs 8-16 and 8-27. [file jvi.00744-24-s0002.tif]

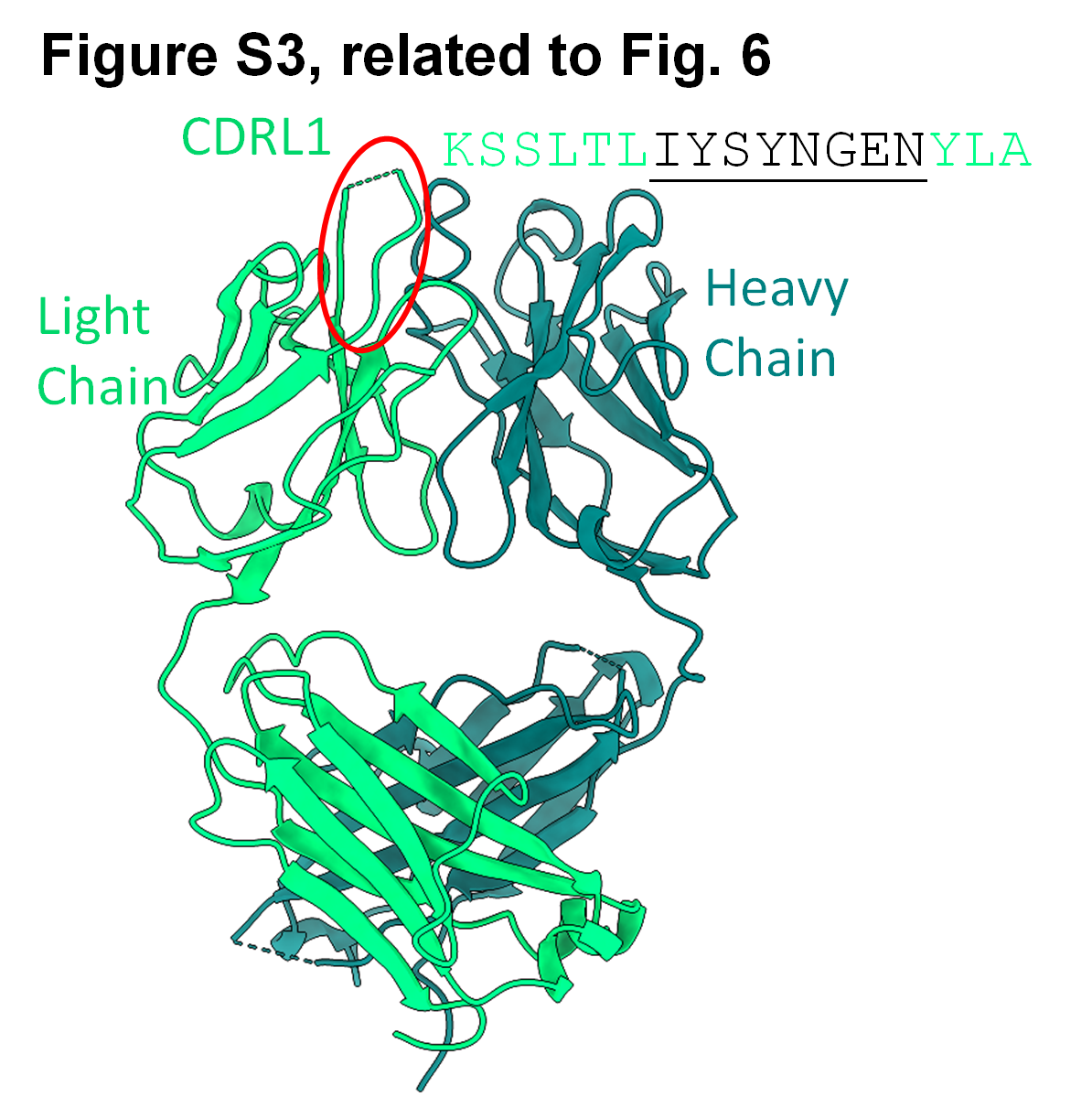

Supplement: Figure S3 — Crystal structure of mAb 8-24. [file jvi.00744-24-s0003.tif]

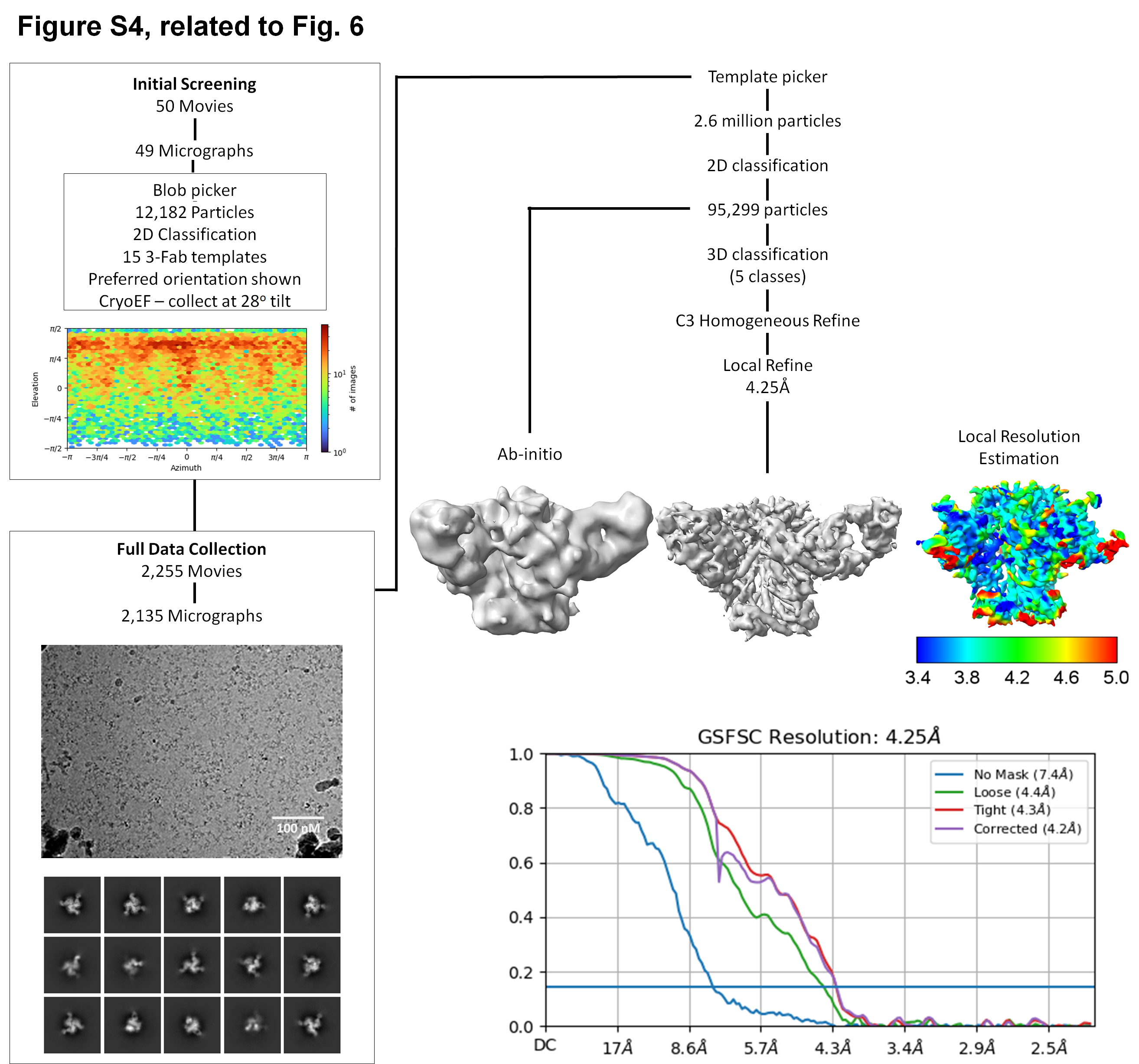

Supplement: Figure S4 — Cryo-EM map processing and resolution pipeline. [file jvi.00744-24-s0004.tif]
