# Supplementary material for: Short CDRL1 in intermediate VRC01-like mAbs is not sufficient to overcome key glycan barriers on HIV-1 Env
Source: J Virol. 2024 Sep 6;98(10):e00744-24. doi: 10.1128/jvi.00744-24 (PMC11495006; doi:10.1128/jvi.00744-24)
Supplement: Supplemental material — Tables S1, S3, S5, and S6; Figure legends. [file jvi.00744-24-s0005.docx]

**SuPPLEMENTAL Figure LEGENDS and TableS**

**Figure S1,** Plasma antibody responses elicited against each indicated Env protein post final boost immunization, related Fig. 1. (A) Each graph represents data for a single animal where each curve represents a time point indicated in the legend (with dotted line of the same color for the KO Env). (B) CD4-BS responses are shown for each protein, calculated based on the end point titer values. Pre-bleed samples from all animals (pool) was used as an internal control in all ELISAs.

**Figure S2,** Estimated probabilities of observed mutations in mAbs 8-16 and 8-27 as described by the ARMADiLLO web server, related to Fig. 4. Top panel shows the heavy chain sequences and bottom panel shows the light chain sequences for the two mAbs.

**Figure S3,** Crystal structure of mAb 8-24, related to Fig. 6. X-ray crystal structure of unbound mAb 8-24 is shown in cartoon representation. The light chain is shown in light green and the heavy chain in teal. The location of the CDRL1 is indicated by the red circle and is partially unresolved. The sequence of the CDRL1 is shown and unresolved residues are underlined.

**Figure S4,** Cryo-EM map processing and resolution pipeline, related to Fig. 6. Representative micrograph and templates used for template picker are shown and boxed. Notable maps obtained during processing are shown. Final sharpened map is shown with local resolution estimation and CryoSPARC GSFSC.

**Table S1**, List of SOSIP immunogens used as a cocktail in the final boost, related to Fig. 1.

| **SOSIP** | **Clade** | **Dose (ug/mice)** |
| --- | --- | --- |
| AMC011 v5.2 | B | 0.909 |
| AMC009 v5.2 | B | 0.909 |
| conH v8.2 | H | 0.909 |
| conB v4.2 | B | 0.909 |
| SFU v5.2 | B | 0.909 |
| AMC008* | B | 0.909 |
| ZM197M* | C | 0.909 |
| DU422 | C | 0.909 |
| ConM | M | 0.909 |
| ConC | C | 0.909 |
| BG505-MD39 | B | 0.909 |

* Indicates lack of N276 NLGS

**Table S2,** HC/LC sequences of the VRC01-like antibodies isolated after the final boost immunization, related to Fig. 2. Amino acid sequences are aligned to the V gene from which they are derived, and CDR are highlighted in red. For sequences where the PCR product did not contain the 5’ end, the sequence is shown beginning at CDR1.

**Table S3,** Information on the VRC01 like mAbs isolated following the final boost immunization with SOSIP cocktail, related to Fig. 3. 17 VRC01-like mAbs were generated from animals following their final immunization, of which 15 were selected for further analysis.

| **Ab name** | **V-GENE and allele-HC** | **J-GENE and allele-HC** | **AA JUNCTION-HC** | **V-GENE and allele-LC** | **J-GENE and allele-LC** | **AA JUNCTION-LC** |
| --- | --- | --- | --- | --- | --- | --- |
| 8-7 | Homsap IGHV1-2*02 | Homsap IGHJ1*01 | CARGKNSDYNWDFQHW | Musmus IGKV12-46*01 | Musmus IGKJ2*01 | CQHFWKF |
| 8-8 | Homsap IGHV1-2*02 | Homsap IGHJ1*01 | CARGKNSDYNWDFQHW | Musmus IGKV8-30*01 | Musmus IGKJ2*01 | CQQYESY |
| 8-9* | Homsap IGHV1-2*02 | Homsap IGHJ1*01 | CARGKNSDYNWDFQHW | Musmus IGKV8-30*01 | Musmus IGKJ2*01 | CQQYYTF |
| 8-10 | Homsap IGHV1-2*02 | Homsap IGHJ1*01 | CARGKDSDYNWDFQHW | Musmus IGKV8-30*01 | Musmus IGKJ2*01 | CQQYETF |
| 8-11* | Homsap IGHV1-2*02 | Homsap IGHJ1*01 | CARGKNSDYNWDFQHW | Musmus IGKV8-30*01 | Musmus IGKJ1*01 | CQQYWTF |
| 8-14 | Homsap IGHV1-2*02 | Homsap IGHJ1*01 | CARGKNSDYNWDFQHW | Musmus IGKV8-30*01 | Musmus IGKJ5*01 | CQQYEYY |
| 8-16 | Homsap IGHV1-2*02 | Homsap IGHJ1*01 | CARGKNSDYNWDFQHW | Musmus IGKV8-30*01 | Musmus IGKJ1*01 | CHQYETF |
| 8-18 | Homsap IGHV1-2*02 | Homsap IGHJ1*01 | CARGKISDYNWDFQHW | Musmus IGKV8-30*01 | Musmus IGKJ1*01 | CQQYYKF |
| 8-19 | Homsap IGHV1-2*02 | Homsap IGHJ1*01 | CARGKDSDYNWDFQHW | Musmus IGKV8-30*01 | Musmus IGKJ1*01 | CQQYYKF |
| 8-20 | Homsap IGHV1-2*02 | Homsap IGHJ1*01 | CARGKTSDYNWDFQHW | Musmus IGKV8-30*01 | Musmus IGKJ5*01 | CQQYYKY |
| 8-21 | Homsap IGHV1-2*02 | Homsap IGHJ1*01 | CARGKDSDYNWDFQHW | Musmus IGKV8-30*01 | Musmus IGKJ2*01 | CQQYEIY |
| 8-22 | Homsap IGHV1-2*02 | Homsap IGHJ1*01 | CARGKKNHYNWDFQHW | Musmus IGKV8-30*01 | Musmus IGKJ2*03 | CQQYEFY |
| 8-23 | Homsap IGHV1-2*02 | Homsap IGHJ1*01 | CARGKDSDYNWDFQHW | Musmus IGKV8-30*01 | Musmus IGKJ5*01 | CQQYYKY |
| 8-24 | Homsap IGHV1-2*02 | Homsap IGHJ1*01 | CARGKNSDYNWDFQHW | Musmus IGKV8-30*01 | Musmus IGKJ2*01 | CQQYEYF |
| 8-25 | Homsap IGHV1-2*02 | Homsap IGHJ1*01 | CARGKDSDYTWDFQHW | Musmus IGKV8-30*01 | Musmus IGKJ2*03 | CQQYYSF |
| 8-26 | Homsap IGHV1-2*02 | Homsap IGHJ1*01 | CARGKDSDYNWDFQHW | Musmus IGKV8-30*01 | Musmus IGKJ1*01 | CQQYYKF |
| 8-27 | Homsap IGHV1-2*02 | Homsap IGHJ1*01 | CARGKNSDYNWDFQHW | Musmus IGKV8-30*01 | Musmus IGKJ5*01 | CQQYEFF |

* Indicates lack of 426c.Mod.Core binding by BLI

**Table S4,** Summary of overall binding properties of the 15 VRC01-like mAbs that were generated from animals following final SOSIP cocktail immunization using Biolayer Interferometry Assay, related to Fig. 3. No binding: (-); Up to 0.1: +/-; 0.1 to 0.5: +; 0.5 to 1: ++; and >1: +++.

**Table S5,** Data collection and refinement statistics for cryo-EM structure, related to Fig. 6.

|  | 426c.WITO.TM.SOSIP with mAb 8-24 |
| --- | --- |
| **Data collection** |  |
| Microscope | Glacios |
| Voltage (kV) | 200 |
| Electron Dose (e-/Å^2^) | 51 |
| Detector | K3 |
| Pixel Size (Å/px) | 1.122 |
| Defocus Range (µm) | -1.0 to -2.5 |
| Collection Tilt (^o^) | 28 |
| Magnification | 36,000x |
| **Reconstruction** |  |
| Software | CryoSPARC v4.4 |
| Selected Micrographs | 3,438 |
| Selected Particles | 23,004 |
| Symmetry | C3 |
| Box Size (px) | 384 |
| Resolution (Å) (FSC_0.143_) | 4.2 |
| **Refinement** |  |
| Map B factor (Å^2^) | 290.0 |
| No. atoms |  |
| Protein | 39936 |
| Water | 0 |
| Ligand | 3618 |
| Mean B-factor (Å) |  |
| Protein | 260.5 |
| Water | 0 |
| Ligand | 298.1 |
| RMS bond length (Å) | 0.005 |
| RMS bond angle (^o^) | 0.613 |
| **Validation** |  |
| MolProbity | 2.35 |
| Clashscore | 19.91 |
| CaBLAM outliers (%) | 3.09 |
| EMRinger | 0.52 |
| Rotamer Outliers (%) | 0.00 |
| Ramachandran |  |
| Favored (%) | 89.93 |
| Disallowed (%) | 0.26 |
| **PDB ID** | 9BGE |
| **EMDB ID** | 44510 |

**Table S6,** Data collection and refinement statistics for crystal structure, related to Fig. 6.

|  | **8-24 His Fab** |  |
| --- | --- | --- |
| **Data collection** |  |  |
| Space group | P 21 21 21 |  |
| Cell dimensions |  |  |
| *a*, *b*, *c* (Å) | 41.87 77.70 266.15 |  |
| *α, β, γ* (°) | 90, 90, 90 |  |
| Resolution (Å) | 44.36 – 2.01 (2.06-2.01) |  |
| *R*_merge_^a^ | 0.099 (0.556) |  |
| <I/σ(I)> | 10.0 (2.4) |  |
| CC_1/2_ | 0.996 (0.857) |  |
| Completeness | 99.1 (98.9) |  |
| Redundancy | 5.8 (5.2) |  |
| **Refinement** |  |  |
| Resolution (Å) | 44.36 – 2.01 (2.082-2.01) |  |
| No. unique reflections | 58514 (5745) |  |
| *R*_work_^b^/*R*_free_^c^ | 22.8/28.0 (28.3/31.6) |  |
| No. atoms | 6644 |  |
| Protein | 6461 |  |
| Water | 143 |  |
| Ligand | 40 |  |
| B-factors (Å^2^) | 33.32 |  |
| Protein | 33.38 |  |
| Water | 29.15 |  |
| Ligand | 37.95 |  |
| RMS bond length (Å) | 0.007 |  |
| RMS bond angle (°) | 0.96 |  |
| **Ramachadran Plot Statistics^d^** | |  |
| Residues | 838 |  |
| Most Favored region | 97.2 |  |
| Allowed Region | 2.80 |  |
| Disallowed Region | 0.00 |  |
| Clashscore | 4.37 |  |
| **PDB ID** | **9B44** |  |

R_merge_ = [∑_h_∑_i_|*I*_h_ – *I*_hi_|/∑_h_∑_i_*I*_hi_] where *I*_h_ is the mean of *I*_hi_ observations of reflection *h*. Numbers in parenthesis represent highest resolution shell. ^b^ R_factor_ and ^c^ R­_free_ = ∑||F_obs_| - |F_calc_|| / ∑|F_obs_| x 100 for 95% of recorded data (R_factor_) or 5% data (R_free_). ^d^ Determined using MolProbity (10.1002/pro.3330)
